# Annotations

**Published:** 1902-06-28

**Authors:** 


### ANNOTATIONS.

### The Steam Organ.

During the summer months those unfortunates who happen to reside near vacant land on which peripatetic entertainers are in the habit of setting up their shows and roundabouts, are exposed to a terrible nuisance from the monotonous and continued screeching of steam organs. In most places the sufferers are without redress. All they can do is either to put up with the nuisance or to leave the neighbourhood, removing to some district which, being more completely built over, offers no vacant spots on which these terrible noise-mills can be erected. Even local authorities are said to have found it impracticable to put a stop to these performances under any clause in the Public Health Act, although we should have thought that the well-known Bishop Auckland case, in which it was decided that anything which interferes with personal comfort is a nuisance, might have been made to cover the use of premises for the making of such noises as are emitted hour after hour from steam organs on certain fair grounds. Of course an application for an injunction may be made, but private individuals not unnaturally hesitate to incur such expense for the general good. The injury which is done to the sick by these instruments of torture is very great, and we are glad to hear that the Fulham Borough Council has lately obtained the sanction of the Home Secretary to a by-law by means of which they will be able to put an effective check upon the use of steam or other organs in connection with any show or performance held in places "adjoining or near to a street" to the "annoyance or disturbance of residents." As the Fulham Council has gone to the trouble of getting this by-law we may presume that they intend to put it in force, and in the name of the sick we thank them for so doing.

### American Pay Patients.

A curious result of the system so widespread in America of making the reception of "pay patients," a sort of business the profits of which go towards the support of the non-pay portion of a hospital, has lately shown itself with some acuteness in New York, and doubtless might be discovered in many other places. Semi-private hospitals, run more on the hotel principle than as pure charities, are far more common in America than with us, although we dare not assert that they are unknown even on this side of the water, and in these institutions the fraud upon the physician which results from receiving patients who are well able to pay full fees is sufficiently apparent. But it is difficult to put a stop to it, for since the hospital authorities are not only repaid for all they expend upon these cases but actually make a profit out of them, they take but little notice of the complaints made by the doctors. So acute has this grievance become that a suit for professional fees has lately been entered by a hospital physician against a hospital patient who from his position was regarded as unfitted to receive the benefits of the charity, and the *Medical Record* of New York commenting on the case says that "The hospital authorities as a rule make no effort to discover the status of a patient, but if he comes with money in hand he is welcome to the best advice and treatment, and the unfortunate medical man too often must grin and bear it." If the doctor complains he is asked to resign, and a more pliable individual is put in his place, while, what is worse still, in making appointments to the staff, ability is no longer the criterion, but those men are chosen who are most likely to keep the pay beds full. Nothing could be worse than all this. For moneyed people to force their way into hospitals, and by dint of their cash payments to make themselves so important as to become the dominant factor in the management of these institutions, would be a great evil, and one entirely opposed to the whole system on which our voluntary charities are supported.

### Aliens and the State.

The evidence given before the Alien Immigration Committee by Mr. Shirley Murphy, Medical Officer of Health for the County of London, must have surprised those people who look upon the foreigner as an unmitigated evil. Mr. Murphy pointed out that there had been a great increase in the foreign population of the district of Stepney during the last ten years. In 1891 the foreign population was 11·3, and in 1901 it was 18·2 per thousand. But in the quinquennium from 1886 to 1890 the death-rate for Stepney was 25 per thousand, while in the period from 1896 to 1900 it was 23·82 per thousand of the population. The influx of foreigners had therefore not affected the health of the neighbourhood injuriously. Moreover, while in London as a whole there had been a falling-off in the birth-rate there had been an increase in Stepney as a whole, and the increase was greatest in Whitechapel and St. George's-in-the-East, the very districts in which there was the largest increase in the alien population. In reply to a question from Sir Kenelm Digby asking for an explanation of this unexpected fact, Mr. Murphy said that he drew from the figures the inference that if the foreign immigration had any effect at all, it was in the direction of the diminution of the death-rate from all causes, and also of the infant death-rate. The conclusion he came to was that the difference was due to the better care the foreigners took of their mode of life. People who saw them daily stated that they were abstemious in their use of alcohol. They took greater care of their children, and led more regular lives than our own people who lived in the same class of house. In face of such testimony as this from a man in Mr. Murphy's position, it must be asked if the agitation against alien immigration is justified. If these aliens are bringing more children into the world, and taking better care of them when they are here than our working people do, can England afford to dismiss or discourage them? The English race has always been able to absorb foreign blood with advantage to itself, and we may ask whether it is not possible that the children of these foreigners may yet become not only good citizens, but good Englishmen, and perhaps in turn good emigrants to our colonies? The question is Will they amalgamate with us as other foreign settlers on our shores have done? and in regard to this the presence of so many Jews among them introduces a great difficulty.

